# Voltammetric Methods in Metallothionein Research

**DOI:** 10.1155/BCA.2005.43

**Published:** 2005

**Authors:** Ivana Šestáková, Tomáš Navrátil

**Affiliations:** J. Heyrovský Institute of Physical Chemistry Academy of Sciences of the Czech Republic Dolejškova 3 Prague 8 182 23 Czech Republic

## Abstract

The application of voltammetric methods using different rates of polarisation on HMDE reveal inert or labile behaviour of Cd- or Zn- complexes in the presence of excessive cadmium or zinc ions in solution. This phenomenon was demonstrated first on the simplest phytochelatin – complex of peptide (γ-Glu-Cys)_2_ Gly with cadmium, later on rabbit liver metallothioneins – Cd_7_ MT in the presence of cadmium and Cd_5_ Zn_2_ MT in the presence of zinc. Voltammetric methods can distinguish between labile and inert complexes present simultaneously and therefore could elucidate their role in reactions of metal ion transfer.

Another method using different rates of polarisation – elimination voltammetry with linear scan – proved that S-tetracoordinated complexes of Cd(II) or Zn(II) in the above-mentioned metallothioneins on HMDE are reduced in the adsorbed state. This implies the possibility of increasing the sensitivity of identification or determination of the above complexes. On carbon composite electrode, similar behaviour of Cd-complexes as on HMDE was observed using differential pulse voltammetry.


### Voltammetric Methods in Metallothionein Research

Ivana Šestáková*, Tomáš Navrátil
*J. Heyrovský Institute of Physical Chemistry, Academy of Sciences of the Czech Republic, Dolejškova 3, 182 23 Prague 8, Czech Republic*

### ABSTRACT

The application of voltammetric methods using different rates of polarisation on HMDE reveal inert or labile behaviour of Cd- or Zn- complexes in the presence of excessive cadmium or zinc ions in solution. This phenomenon was demonstrated first on the simplest phytochelatin – complex of peptide ($\gamma$-Glu-Cys)$_2$Gly with cadmium, later on rabbit liver metallothioneins – $Cd_7MT$ in the presence of cadmium and $Cd_5Zn_2MT$ in the presence of zinc. Voltammetric methods can distinguish between labile and inert complexes present simultaneously and therefore could elucidate their role in reactions of metal ion transfer.

Another method using different rates of polarisation – elimination voltammetry with linear scan – proved that S-tetracoordinated complexes of Cd(II) or Zn(II) in the above-mentioned metallothioneins on HMDE are reduced in the adsorbed state. This implies the possibility of increasing the sensitivity of identification or determination of the above complexes. On carbon composite electrode, similar behaviour of Cd-complexes as on HMDE was observed using differential pulse voltammetry.

### INTRODUCTION

Metallothioneins (MT) are low molecular weight proteins (6-7 kDa), characterised by high cysteine content and ability to bind metals like zinc, cadmium, copper, mercury. MT occur in many living species, e.g. vertebrates, crustaceans, mussels, fungi, plants /1/. In 1985, simpler polypeptides with similar properties were discovered in certain plants, named today as MT family 99 or phytochelatins /2, 3/. Metallothioneins are considered to be important in homeostatic control, metabolism and detoxification of several essential or toxic trace metals, and their role is examined in connection with oxidative stress and cancerogenesis /4 /.

The presence of electroactive groups – cysteinyl residues and metals bound through metal-thiolate bonds in MT molecule – is the reason for application of electrochemical methods in metallothionein research. Apart from the determination of total metallothionein concentration by modified Brdička reaction /5-9/, voltammetric methods using hanging mercury drop electrode are employed for characterisation of

*Corresponding author. *E-mail address:* sestakov@jh-inst.cas.cz (I. Šestáková).
Tel.: +420-26605-3875, fax: +420-28658-2307

metallothioneins with respect to bound metal. The studies performed up to now deal with mammalian metallothioneins, mainly with rabbit liver MT, where structural arrangement and tetracoordination of metals by four sulphur atoms has been documented by NMR and XR studies. 61 amino acids, 20 of them being cysteine, form the molecule of rabbit liver $Cd_5Zn_2MT$, which is arranged in two clusters. The cluster α binds four Cd (II), whereas cluster β binds one Cd(II) and two Zn (II) /10, 11/.

Besides reduction of complexed Cd(II) and Zn(II), signals due to the formation and reduction of mercury compound at potential range more positive than –0.6 V ($Ag/AgCl_{sat}$) could be recognised at pH 8.5–9 using cyclic voltammetry on hanging mercury drop electrode. The formation of mercury compound is blocked, when mercury surface is covered by compact triphenylphosphine oxide or tripiperidinophosphine oxide film /12/.

On the other hand, the signal of mercury compound can be used for characterisation of MT sample in solution. Under conditions of lack of metal, free -SH (or S-S after oxidation) will give a signal due to the formation and reduction of mercury compound in the same range as the apometallothionein does, $Ep_a \sim Ep_c$ – 0.725 V (pH 8.5 – 9, $Ag/AgCl_{sat}$) /12/.

The addition of cadmium ions or zinc ions into solution with MT is connected with the appearance of reduction peaks in less negative potentials region than that where the reduction of complex with tetracoordinated metal occurs. This would suggest the existence of a different and less stable complex. Even when more detailed studies of the dependence on the added metal concentration or on pH were performed /13-17/, no unambiguous explanation could be made. In the present study, cyclic voltammetry at different scan rates was used to examine inert or labile behaviour of Cd-complexes of synthetically prepared simplest phytochelatin-peptide $(\gamma\text{-Glu-Cys})_2Gly$ and complexes of Cd and Zn in $Cd_7MT$ and $Cd_5Zn_2MT$. For selected concentrations, the method of elimination voltammetry with linear scan was applied, which can bring more information about the electrode process involved /18-21/.

### EXPERIMENTAL

### Apparatus

Voltammetric measurements were performed using PC-ETP analyser (Polaro Sensors, Czech Republic), mostly using Polar 5.0 software /22/. As a working electrode, a pen-type mercury electrode, drop surface area 0.013 $mm^2$, was used. $Ag/AgCl_{sat.}$ was a reference and Pt wire an auxiliary electrode. For deoxygenation of solutions prior to the measurements, purified nitrogen was applied and passed above the solution during measurements.

Carbon composite paste electrode (CCPE) had a 2 mm disc surface diameter and was prepared by hand-mixing of graphite powder with paraffin oil and 10% w.w. of silica gel. The paste was packed into a Teflon electrode body with a stainless steel piston, serving for renewing of the paste surface as well as for an electric contact. The new surface was smoothed on card paper and pretreated prior to each measurement by applying potential +1.4 V for 10 s in a stirred solution of the measured sample.

### Reagents and procedures

The cadmium metallothionein (lot 56H9500, cadmium content 5.9%, zinc content 0.5%) and cadmium-zinc metallothionein (lot 80K7013, cadmium content 7.9%, zinc content 1.4%) were products of Sigma; a freshly prepared solution in borate buffer pH 8.5 was used for measurements. Double distilled water (quartz apparatus) and Suprapure grade chemicals $Na_2B_4O_7$, $3CdSO_4$ $8H_2O$ and $HNO_3$ 65% (Merck) were used. Cysteine (free base, Sigma) Metalfix Chelosolve (Na+ form, 40-80 μm, Fluka), medicinal paraffin oil, silica gel 30 μm (Lachema) and graphite powder 99.9% (Fluka) were used.

$(\gamma$-Glu-Cys$)_2$Gly was synthesised using the Merrifield method /23/ in the Institute of Organic Chemistry and Biochemistry, AS CR, Prague. For measurements with HMDE, freshly prepared solutions of peptide in borate buffer pH 8.5 were used.

The absence of traces of Pb, Cd or Cu in buffer solutions was proved by anodic stripping voltammetry on HMDE (detection limits 1 x $10^{-10}$ mol.$L^{-1}$ for Cd and Pb, and 1 x $10^{-9}$ mol.$L^{-1}$ for Cu, using 360s of deposition at –850 mV in acidic solution).

A solution of CdMT was "saturated" prior to measurement with cadmium in order to exclude the presence of free SH groups. In a deoxygenated borate buffer pH 8.5, 1x$10^{-5}$ M $Cd^{2+}$ was added to 3.3x$10^{-5}$ M CdMT. A column of Metalfix Chelosolve (80 x 12 mm) was used to remove excessive $Cd^{2+}$; differential pulse cathodic stripping voltammetry was used to confirm the saturation /24/.

### Data treatment

Elimination voltammetry with liner scan (EVLS) has been applied in selected cases of solutions of MT or peptide with cadmium ions, as this method can supply more detailed information about the process on the electrode. The EVLS theory /25/ has been developed on the presumption that the total current can be expressed as a sum of particular currents $I_j$

$$I = \sum I_j \qquad I = I_d + I_c + I_k + \dots$$

and that it is possible to express each particular current in the form of two independent functions

$$I_j = W_j(v)Y_j(E)$$

where the Y(E) functions are specific for every current and W(v) functions are dependent on the scan rate v, e.g.

| | | |
|---|---|---|
| e.g. | $I_d = v^{1/2}Y_d(E)$ | diffusion current |
| | $I_k = v^0 Y_k(E)$ | kinetic current |
| | $I_c = v^1 Y_c(E)$ | charging current |

The current function is then constructed

$$f(I) = \sum_k a_k I(v_k)$$

and a set of m equations for m scan rates is solved. In this way, some currents can be conserved and some eliminated when $f(I_j) = 0$.

As in MT reduction peaks are diffusion controlled, we used calculations for current function in cases where diffusion current is conserved and kinetic and charging current is eliminated:

$f(I_d) = 17.4857\,I - 11.657\,I_{1/2} - 5.8284\,I_2$ equation 23 in /25/, or

$f(I_d) = 43.213\,I - 48.041\,I_{1/2} - 11.657\,I_2 + 16.485\,I_{1/4}$ equation 31 in /25/

In theoretical simulation, it can be distinguished if the transported species is adsorbed before reduction or not. In case of adsorption, characteristic peak- counterpeak form is obtained for the above mentioned $f(I_d)$ current function /26/.

For each EVLS measurement, set of DC-voltammograms with different rates of polarisation ($v_{ref}$, v1/2, v1/4, 2v) was recorded and a digital set of current values was used for calculation.

### RESULTS AND DISCUSSION

As illustrated in Figure 1, the reduction peak of Cd-complex (A) in CdMT or CdZnMT appears at about –0.85 V, that of Zn-complex (B) at about –1.25 V (pH 8.5 – 9). Both complexes behave at cyclic voltammetry as inert – the height of the cathodic peaks (A, B) as well as of the anodic peaks (A′, B′)

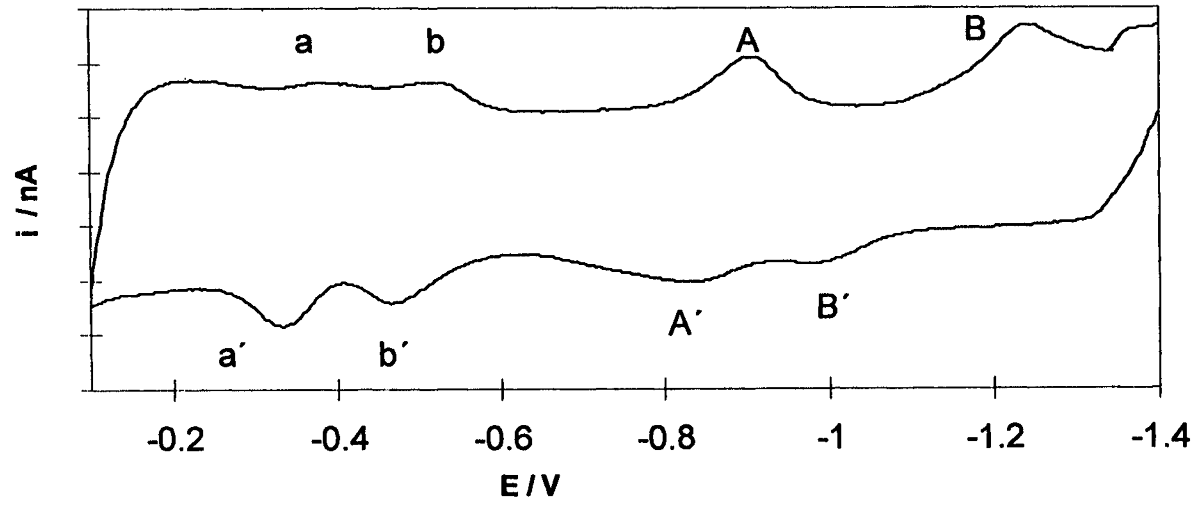

**Fig. 1:** Cyclic voltammogram of 6 x $10^{-6}$ M Cd,Zn-MT in borate buffer pH 9.1, scan rate 500 $mV.s^{-1}$, $Ag/AgCl_{sat.}$ ref. A-reduction peak of Cd-complex , B-reduction peak of Zn-complex, A′, B′ – peaks corresponding to the formation of Cd- and Zn-complex, a, a′, b, b′ – peaks corresponding to mercury compounds increases with increasing rate of polarisation. In agreement with data from NMR and XR that all metal ions in rabbit MT structure are coordinated by four sulphur atoms, only one signal is observed for each metal, as they are in equivalent chemical environments.

The application of the EVLS method shows that the reduction of Cd-complex as well as Zn- complex of Cd, Zn-MT on HMDE takes place in an adsorbed state. Similarly, the peak-counterpeak shape of current function $f(I_d)$ is obtained in EVLS of CdMT (Fig.2).

For further study of voltammetric behaviour in the presence of excessive ions, where more positive reduction peaks than those corresponding to the reduction of tetracoordinated complexes are observed, a model system was chosen – peptide ($\gamma$-Glu-Cys)$_2$Gly with cadmium. The reason is that this is the simplest system, where using EXAFS, similar coordination of Cd(II) as in rabbit liver metallothionen was confirmed /27, 28/. Due to the occurrence of overlapping signals /29/, voltammetric measurements were performed in order to allow application of multivariate curve resolution method by alternating least-squares (MCR-ALS). Such a procedure was successful in solution of complexation of simple molecules such as glutathione with cadmium /30/ or zinc /31/ and also glutathione fragments Cys-Gly and $\gamma$-Glu-Cys with cadmium /32/.

For the system ($\gamma$-Glu-Cys)$_2$Gly and Cd(II) after application MCR-ALS method, different reduction signals were resolved, corresponding to Cd(II) coordinated by 1, 2 or 4 sulphur atoms /33/. From the concentration profile plot the stoichiometries and possible structures of complexes could be deduced. Corresponding complexes could then be prepared in solution at a given ratio of peptide to cadmium ions.

For the complex formed when $Cd^{2+}$ is added to peptide at ratio Cd/P equal to 0.2, the structure with Cd(II)

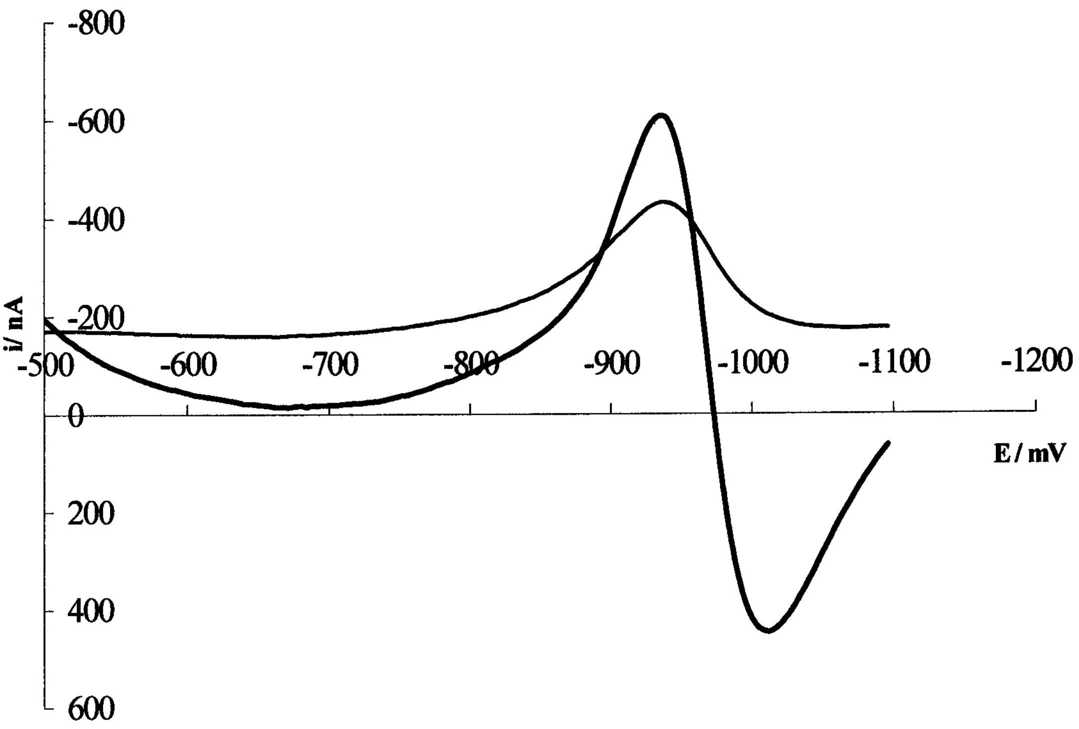

(a)

continued...

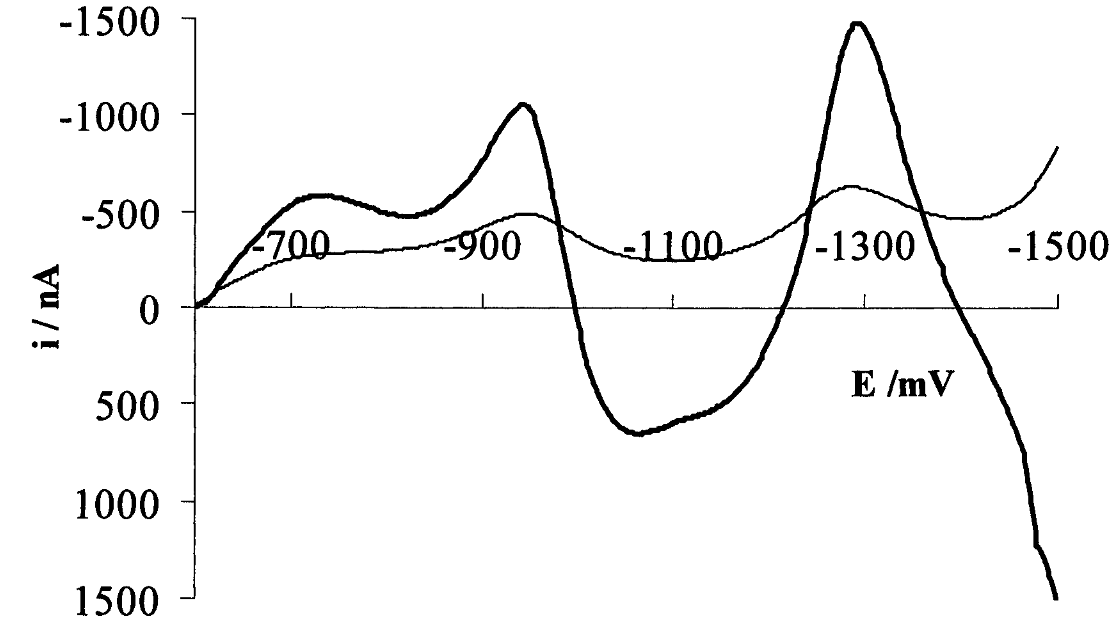

(b)

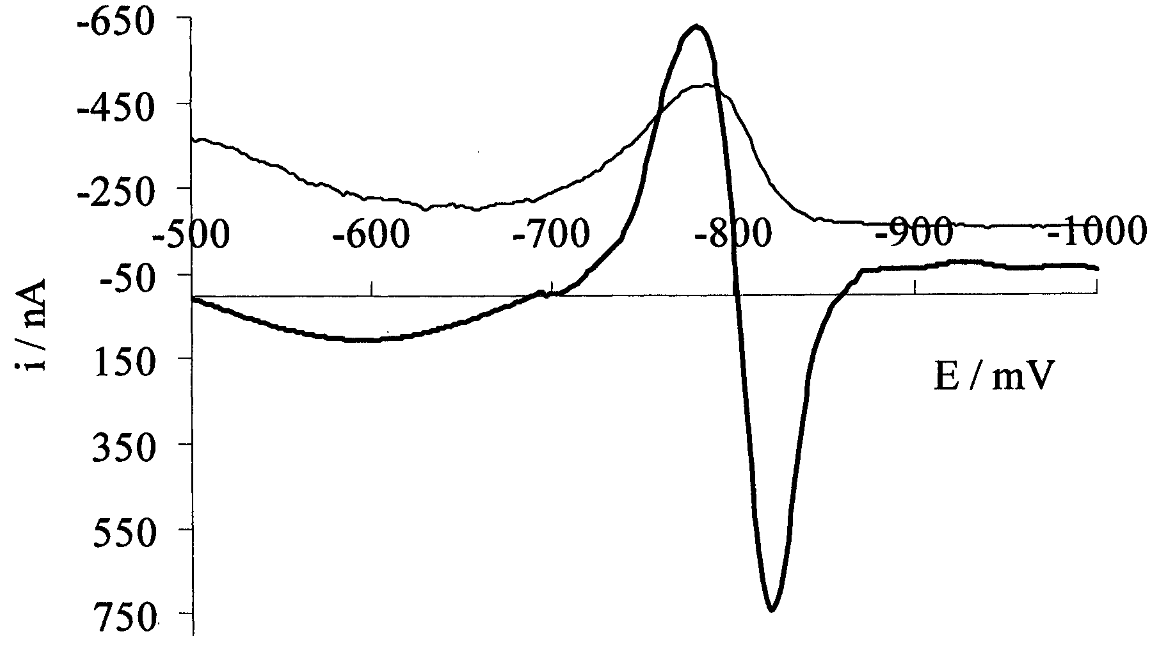

(c)

**Fig. 2:** Elimination voltammetry with linear scan. a – 3.2 x $10^{-6}$ M $Cd_7MT$ (– $v_{ref}$ = 400 mV/s, — $f(I_d)$ = eq. 31), b – 1 x $10^{-6}$ M $Cd_5Zn_2MT$ (– $v_{ref}$ = 800 mV/s, — $f(I_d)$ = eq.23), c – 2 x $10^{-5}$ M peptide (γ-Glu-Cys)$_2$Gly + 4 x $10^{-6}$ $Cd^{2+}$ (– $v_{ref}$ = 400 mV/s, — $f(I_d)$ = eq. 23).

coordinated by four sulphur atoms is implied from stoichiometry. At the above Cd to peptide ratio, tetracoordinated complex is the only one formed (CdP) and its behaviour can be studied by cyclic voltammetry. The voltammograms recorded at different rates of polarisation demonstrated inert behaviour of this complex, similarly to complex of tetracoordinated cadmium in rabbit liver CdMT or Cd,Zn MT exhibited. The application of EVLS method shows that this Cd-peptide complex is also reduced in the adsorbed state (Fig.2c).

With further additions of cadmium ions, complexes where two or finally one sulphur atoms are involved in Cd(II) coordination (complex CdP′, Figure 3), labile behaviour of such complex is observed in cyclic voltammetry. Examining the dependence on the rate of polarisation, differentiation between labile and inert behaviour appears. As may be seen from Figure 4, the labile complex could be identified only at certain values of polarisation rates – at low rates, the metal falls out and a peak of uncomplexed metal could be seen. At high scan rates – only inert complex is formed as there is no time for rearrangement leading to the labile complex.

Using a broad range of rates of polarisation, the appropriate scan rates values were found when labile behaviour of Cd-complex of CdMT in presence of cadmium ions appears. Similarly, labile behaviour of Zn complex after addition of zinc ions to Cd,Zn-MT was confirmed (Fig.5).

Beside mercury electrode, carbon electrodes were examined. Composite carbon paste electrode (10% $SiO_2$) has been proved as suitable for the study of cadmium complexes /24/. After accumulation at potential where Cd-complex is reduced, the oxidation peaks could be observed when potential is changing towards positive values at differential pulse voltammetry. Beside the peak of oxidation Cd(0) → $Cd^{2+}$, two peaks in range −250 to +250 mV were observed for CdMT, which means formation of two Cd(II) complexes, similarly as on HMDE. For the peptide ($\gamma$-Glu-Cys)$_2$Gly and Cd(II) at large excess of peptide, oxidation peak at +150 mV was observed. This peak, as well as peak at +190 mV for CdMT we consider as formation of tetracoordinated complex. (Fig. 6). The oxidation peak at region +150 to + 220 mV we observed earlier /12/

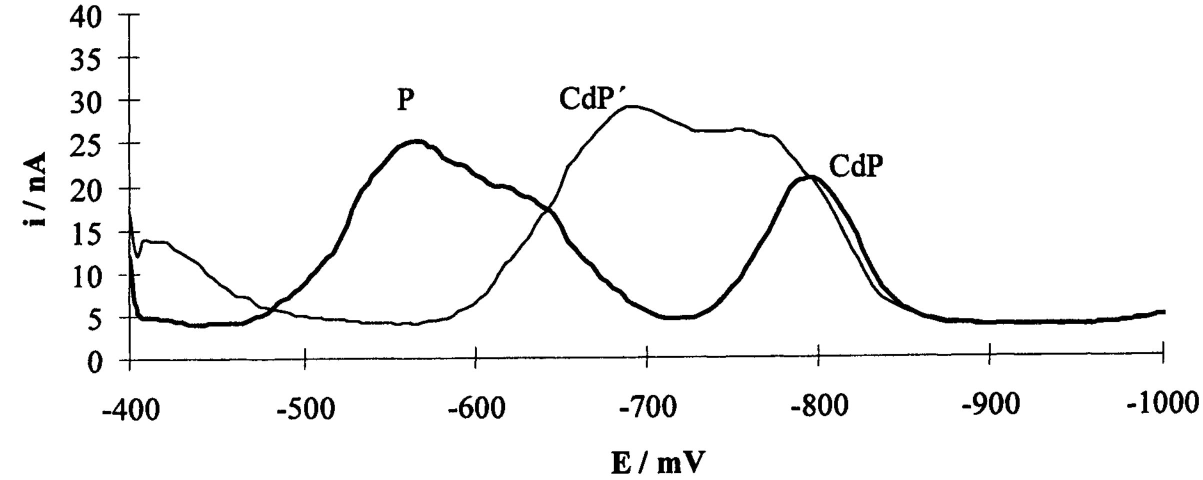

**Fig. 3:** Additions of $Cd^{2+}$ to 1 x $10^{-5}$ M peptide ($\gamma$-Glu-Cys)$_2$ Gly, borate buffer pH 8.5, differential pulse voltammetry on HMDE (P- reduction of Hg-peptide, CdP and CdP′ – reduction of Cd(II)-peptide complexes).

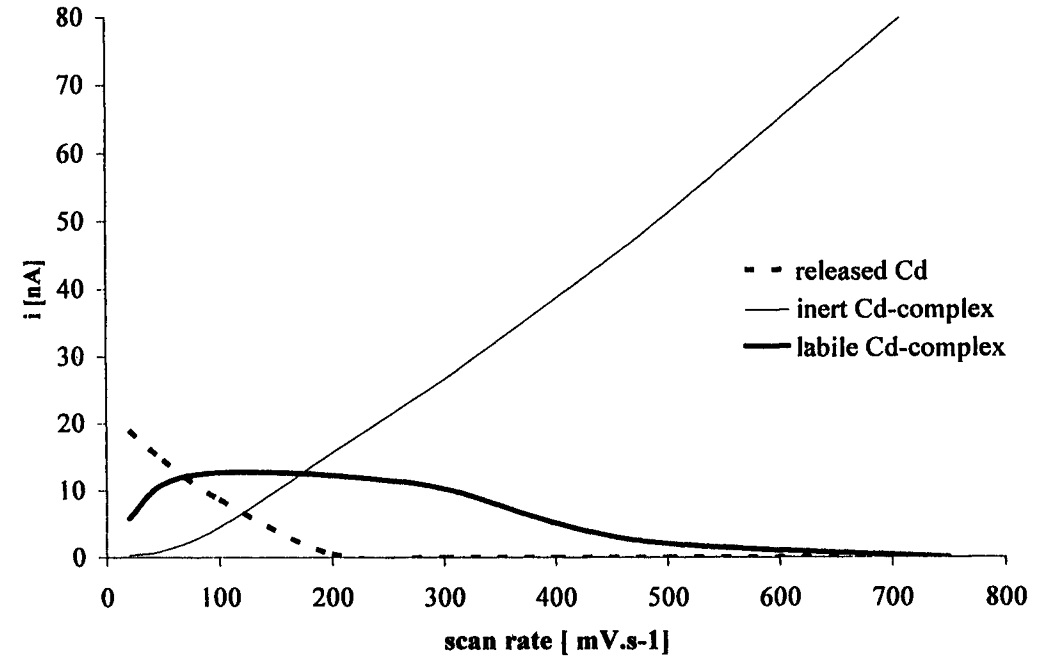

**Fig. 4:** 5 x $10^{-6}$ M peptide (γ-Glu-Cys)$_2$ Gly and 1 x $10^{-5}$ M $Cd^{2+}$ , borate buffer pH 8.5. The dependence of anodic-peak- height on rate of polarisation in cyclic voltammetry on HMDE.

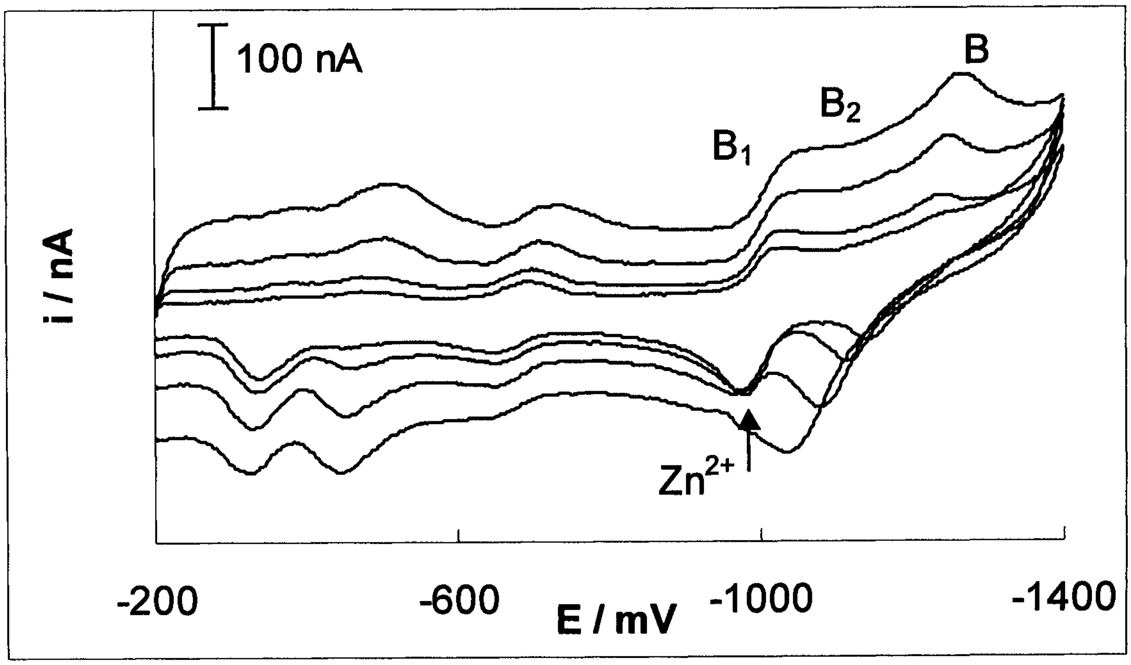

**Fig. 5:** Labile behaviour of Zn complex (peaks $B_1$ and $B_2$) in 1 x $10^{-6}$ M $Cd_5Zn_2MT$ + 1 x $10^{-6}$ M $Zn^{2+}$, borate buffer pH 8.5, CV on HMDE from -200 mV, scan rate – mV/s: 50, 100, 200, 400.

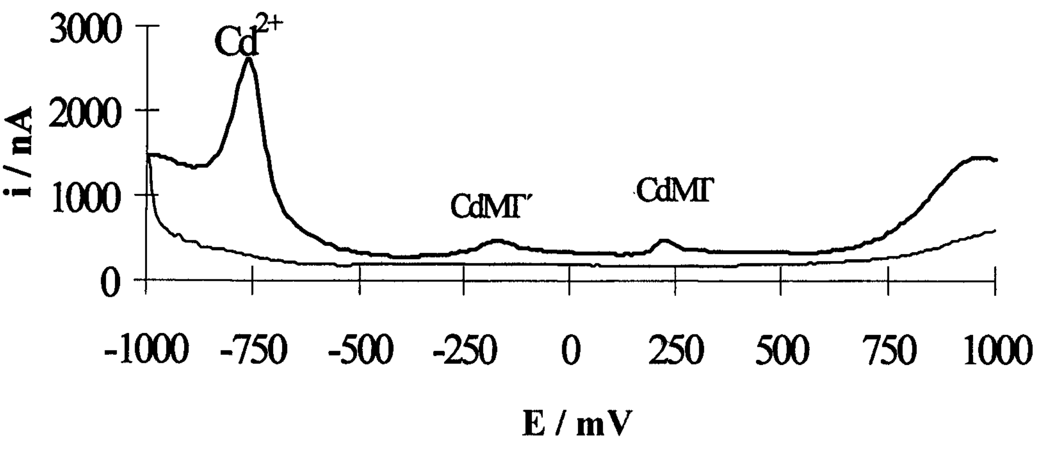

(a)

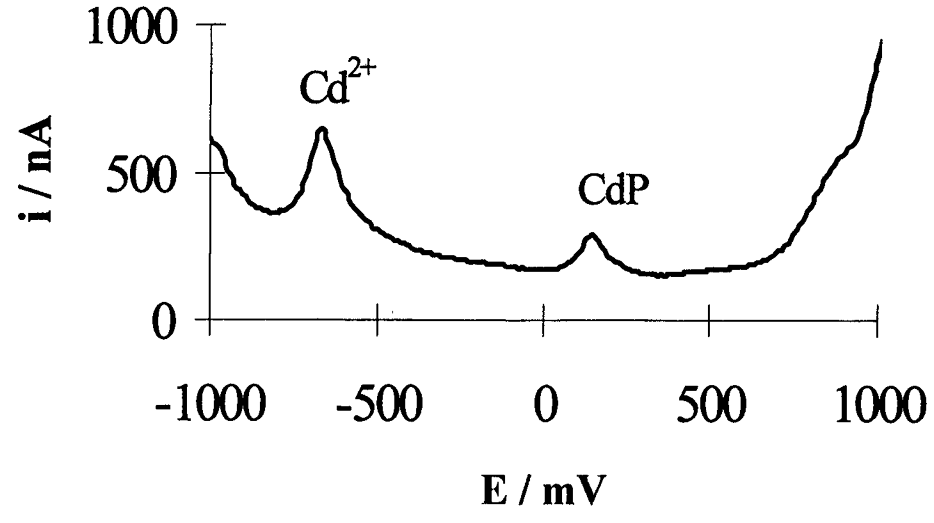

(b)

**Fig. 6:** Differential pulse voltammetry on carbon composite electrode, borate buffer pH 8.5, scan from – 1000 to + 1000 mV, scan rate 20 mV/s.
a – 5.2 x $10^{-6}$ M CdMT, DPV after 120s accumulation at –1400 mV,
b – 2 x $10^{-5}$ M peptide (γ-Glu-Cys)$_2$ Gly, 2 x $10^{-6}$ M $Cd^{2+}$, DPV after 120s accumulation at –1400 mV.

using cyclic voltammetry in case of adsorbed layer of CdMT on carbon composite electrode. Apometallothionein is oxidized /12/ at potential +0.8 V (1x$10^{-3}$ M Apo MT, pH 8.3), peptide (γ-Glu-Cys)$_2$Gly at about similar potential. Therefore, no interference due to the presence of free –SH groups are encountered. Nevertheless, due to only slow scan rates available in DPV method, no differentiation between inert and labile behaviour is possible.

Complexes of higher stoichiometry than $Cd_7MT$ were reported by CD /34, 35/ and ESI-MS /36/, but only for cadmium complexes, not for excessive zinc, even when in parallel voltammetric measurement such complexes were identified /37/. The reason could be labile behaviour of such complexes. Therefore voltammetry, which can distinguish between inert and labile complex present simultaneously, seems to be a suitable method for estimation of the role of inert and labile zinc complexes in reactions of metal ion transfer.

### CONCLUSIONS

Using the complexation model of peptide ($\gamma$-Glu-Cys)$_2$Gly with cadmium, where stoichiometries of complexes with respect to the number of sulphur atoms involved were determined using voltammetry in combination with MCR-ALS method, solutions of complexes at selected peptide to cadmium ratio were prepared and studied by voltammetric methods. Using cyclic voltammetry on hanging mercury electrode, inert and labile behaviour of complexes was found. Inert behaviour exhibits the complex where cadmium is coordinated by four sulphur atoms, as found by EXAFS. Labile complexes are formed in higher metal ions concentrations, where two or one sulphur atoms are involved in coordination. Voltammetric methods with low rate of polarisation can detect both types of complexes, whereas only inert complexes are seen by fast methods (high rate of polarisation). Conditions were found when labile behaviour of $Cd_7MT$ at excessive cadmium ions concentration or $Cd_5Zn_2MT$ in excessive zinc ion concentration is pronounced in voltammetric measurement. This brings the possibility of voltammetric methods to establish the role of inert and labile types of complexes in reactions of metal ion transfer.

Application of EVLS proved that all inert complexes studied – inert complexes of Cd(II) which are present in $Cd_7MT$, $Cd_5Zn_2MT$ as well as inert Cd-complex of ($\gamma$-Glu-Cys)$_2$Gly –on HMDE are reduced in the adsorbed state. The same is valid for reduction of inert Zn(II) complex in $Cd_5Zn_2MT$. This finding can be further exploited in application of adsorptive voltammetry in cases where higher sensitivity is required. The resolution of overlapped peak can be also easily solved with the application of EVLS.

### ACKNOWLEDGEMENTS

This research has been partly supported by grants GA ČR 525/02/0301 and MŠMT – COST- OC D21.002.

### REFERENCES

1. C. D. Klaassens (Ed.), *Metallothionein IV*, Birkhauser, Basel, 1999.
2. W. E. Rauser, *Plant. Physiol.*, **109**, 1141(1995).
3. M. H. Zenk, *Gene*, 179 (1996).
4. K. T. Suzuki, N. Imura, M. Kimura (Eds.), *Metallothionein III, Biological Roles and Medical Implications.*, Birkhauser, Basel, 1993.

5. R. W. Olafson, R. Sim, *Anal. Biochemistry*, **100**, 343 (1979).
6. B. Raspor, *J. Electroanal. Chem.*, **503**, 159 (2001)
7. M. Erk, D. Ivankovic, B. Raspor, J. Pavicic, *Talanta*, **57**, 1211 (2002)
8. B. Raspor, M. Paic, M. Erk, *Talanta*, **55**, 109 (2001)
9. R. Kizek, L. Trnková, E. Paleček, *Anal. Chem.*, **73**, 4801 (2001)
10. M. J. Stillman, C. F. Shaw III, K. T. Suzuki (eds.), *Metallothioneins. Synthesis, Structure and Properties of Metallothioneins, Phytochelatins and Metal-Thiolate Complexes.* VCH, New York, 1992.
11. M. J. Stillman, *Coord. Chem. Rev.*, **144**, 461 (1955)
12. M. Fedurco, I. Šestáková, *Bioelectrochem. Bioenerg.*, **40**, 223 (1996)
13. I. Šestáková, D. Miholová, H. Vodičková, P. Mader, *Electroanalysis*, **7**, 237 (1995)
14. C. Ruiz, J. Mendieta, A. R. Rodríguez, *Anal. Chim. Acta*, **305**, 285 (1995)
15. Ch. Harlyk, O. Nieto, G. Bordin, A. R. Rodríguez, *J. Electroanal. Chem.*, **458**, 199 (1998)
16. M. Dabrio, A. R. Rodríguez, *Anal. Chim. Acta*, **385**, 295 (1999)
17. A. R. Rodríguez, M. Esteban, *Cell Mol. Biol.*, **46**, 237 (2000)
18. L. Trnková, O. Dračka, *J. Electroanal. Chem.*, **413**, 123 (1996)
19. L. Trnková, J. Friml, O. Dračka, *Bioelectrochemistry*, **54**, 131 (2001)
20. L. Trnková, R. Kizek, O. Dračka, *Bioelectrochemistry*, **55**, 131 (2002)
21. L. Trnková, *Talanta*, **56**, 887 (2002)
22. M. Dřevínek, F. Trojánek, *Chem. Listy*, **95**, 231 (2001)
23. B. Merrifield, Solid phase peptide synthesis, in: B. Gutte (Ed.) *Peptides: Synthesis, Structures and Applications,* Academic Press, San Diego, 1995, pp.93-169.
24. I. Šestáková, M. Kopanica, L. Havran, E. Paleček, *Electroanalysis*, **12**, 100 (2000)
25. O. Dračka, *J. Electroanal. Chem.*, **402**, 19 (1996)
26. L. Trnková, R. Kizek, O. Dračka, *Electroanalysis*, **12**, 905 (2000)
27. H. Strasdeit, A. K. Duhme, R. Kneer, M. H. Zenk, Ch. Hermes, H. F. Nolting, *J. Chem. Soc., Chem. Commun*, 1129 (1991)
28. I. Pickering, R. G. Prince, G. A. George, *et.al.*, *Biochim. Biophys. Acta*, **1429**, 351 (1999)
29. I. Šestáková, P. Mader, *Cell. Mol. Biol.*, **46**, 257 (2000)
30. M. S. Díaz- Cruz, J. Mendieta, R. Tauler, M. Esteban, *J. Inorg. Biochem.*, **66**, 29 (1997)
31. M. S. Díaz-Cruz, J. Mendieta, A. Monjonell, R. Tauler, M. Esteban, *J. Inorg. Biochem.*, **70**, 91 (1998)
32. B. H. Cruz-Vásquez, J. M. Díaz -Cruz, Ch. Arino, M. Esteban, R. Tauler, *Analyst*, **127**, 401(2002)
33. B. H. Cruz, J. M. Díaz-Cruz, I. Šestáková, J. Velek, Ch. Arino, M. Esteban, *J. Electroanal. Chem.*, **520**, 111 (2002)
34. N. Cols, N. Romero-Isart, M. Capdevila, B. Oliva, P. Gonzáles-Duarte, S. Atrian, *J. Inorg. Biochem.*, **68**, 157 (1997)
35. M. Capdevilla, N. Cols, N. Romero, R. Gonzáles-Duarte, *Cell. Mol. Life Sci.*, **53**, 681 (1997)
36. M. Dabrio, G. Van Vyncht, G. Bordin, A. R. Rodríguez, *Anal. Chim. Acta*, **435**, 319 (2001)
37. M. S. Díaz- Cruz, M. J. López, J. M. Díaz -Cruz, M. Esteban, *J. Electroanal. Chem.*, **532**, 114 (2002)

